# Impact of an electronic alert on prescription patterns of meropenem, voriconazole and caspofungin

**DOI:** 10.1186/s12879-021-06980-1

**Published:** 2021-12-20

**Authors:** Lionel Chok, Katharina Kusejko, Nadia Eberhard, Sandra E. Chaudron, Dirk Saleschus, Claudine Kocher, Roger D. Kouyos, Rainer Weber, Stefan P. Kuster

**Affiliations:** Division of Infectious Diseases and Hospital Epidemiology, University Hospital and University of Zurich, Rämistrasse 100, Zurich, Switzerland

**Keywords:** Antimicrobial stewardship, Antimicrobial consumption, Antimicrobial prescriptions, Antibiotic consumption, Electronic alert, Self-stewardship

## Abstract

**Background:**

Antimicrobial stewardship programs promote the appropriate use of antimicrobial substances through the implementation of evidence-based, active and passive interventions. We analyzed the effect of a computer-assisted intervention on antimicrobial use in a tertiary care hospital.

**Methods:**

Between 2011 and 2016 we introduced an electronic alert for patients being prescribed meropenem, voriconazole and caspofungin. At prescription and at day 3 of treatment, physicians were informed about the risk related to these antimicrobial substances by an electronic alert in the medical records. Physicians were invited to revoke or confirm the prescription and to contact the infectious disease (ID) team. Using interrupted time series regression, the days of therapy (DOTs) and the number of prescriptions before and after the intervention were compared.

**Results:**

We counted 64,281 DOTs for 5549 prescriptions during 4100 hospital stays. Overall, the DOTs decreased continuously over time. An additional benefit of the alert could not be observed. Similarly, the number of prescriptions decreased over time, without significant effect of the intervention. When considering the three drugs separately, the alert impacted the duration (change in slope of DOTs/1000 bed days; *P* = 0.0017) as well as the number of prescriptions (change in slope of prescriptions/1000 bed days; *P* < 0.001) of voriconazole only.

**Conclusions:**

The introduction of the alert lowered prescriptions of voriconazole only. Thus, self-stewardship alone seems to have a limited impact on electronic prescriptions of anti-infective substances. Additional measures such as face-to-face prompting with ID physicians or audit and feedback are indispensable to optimize antimicrobial use.

## Background

Antibiotic overuse in human and veterinary medicine has led to an increase in resistant bacteria [1–4]. Antimicrobial stewardship programs (ASPs) promote the optimal use of anti-infective drugs, reduce unnecessary antimicrobial prescriptions, resistance pressure in the hospital, drug-related side effects and improve patient outcomes [5–8]. ASPs consist of active, usually prospective audits with patient-based interventions and passive interventions, including non-patient-based educational measures and hospital guidelines [9, 10]. Passive strategies seem to be most effective when combined with active interventions. Interventions may be drug-based or disease-based.

Resources allocated to ASPs may often be limited. Individual disease-based support for antimicrobial decision making by an infectious disease (ID) specialist or a pharmacist with antimicrobial stewardship training is time-consuming and costly. Computer-based surveillance and clinical decision support systems have been shown to improve the use of antimicrobials and thus may be part of ASPs [9]. Algorithms providing assistance on antibiotic decision-making may thus allow a broad and cost-saving implementation of ASP interventions.

The Guidelines of the Infectious Diseases Society of America (IDSA) and the Society for Healthcare Epidemiology of America (SHEA) on ASPs support interventions encouraging routine review of the appropriateness of antibiotic therapy by the prescriber itself [8].

Similar to trends observed in Europe, consumption of carbapenems in Swiss hospitals increased by almost 85% between 2004 and 2010 [11]. The emergence of carbapenem-resistant Enterobacterales is thought to be strongly linked to antibiotic overuse and the resulting selection pressure in humans, apart from insufficient infection control measures and antibiotic consumption in agriculture [12–15].

On July 1st, 2011, the Department of Infectious Diseases at the University Hospital Zurich implemented a computer-assisted drug-based intervention on all acute care units which aimed to optimize the use of the broad-spectrum and costly antimicrobials meropenem, voriconazole and caspofungin. Meropenem is a last resort substance and had the broadest spectrum of activity at the time. It accounted for 29% of the total antibiotic cost and was the third most used antibiotic (4.63 defined daily doses (DDD)/100 bed days; ranked after amoxicillin/clavulanic acid and ciprofloxacin, but before piperacillin/tazobactam; non-published data) in our institution in 2009. Caspofungin and voriconazole accounted for 25% and respectively 6% of the total anti-infective costs but only for 2% and 1% of the anti-infective use, respectively, in our institution in 2009. During the electronic prescription of one of these substances, a pop-up window appeared, which highlighted the restrictions and indications associated with these antimicrobials.

The objectives of this study were to assess the effect of the electronic reminder on the number of days of therapy (DOTs) of meropenem, caspofungin and voriconazole, and whether the number of prescriptions of these antimicrobials was affected by the intervention, respectively.

## Methods

### Study design, setting and ethical considerations

The prospective, single-centre, before-and-after study was conducted at the University Hospital Zurich, a reference centre for 1.5 million inhabitants and one of the largest Swiss tertiary care hospitals with 950 beds. The Department of Infectious Diseases and Hospital Epidemiology designed and planned the intervention. The Medical Data Management Team implemented the alert in the electronic patient charts (KISIM™, Cistec®, Switzerland).

This study complies with the national legal and regulatory requirements and the current version of the Declaration of Helsinki and has been approved by the Canton Ethics Committee (Kantonale Ethikkommission Zurich, Switzerland, KEK-ZH-Nr 2011-0031/1 and PB_2018-00032).

### Intervention

**Fig. 1:**
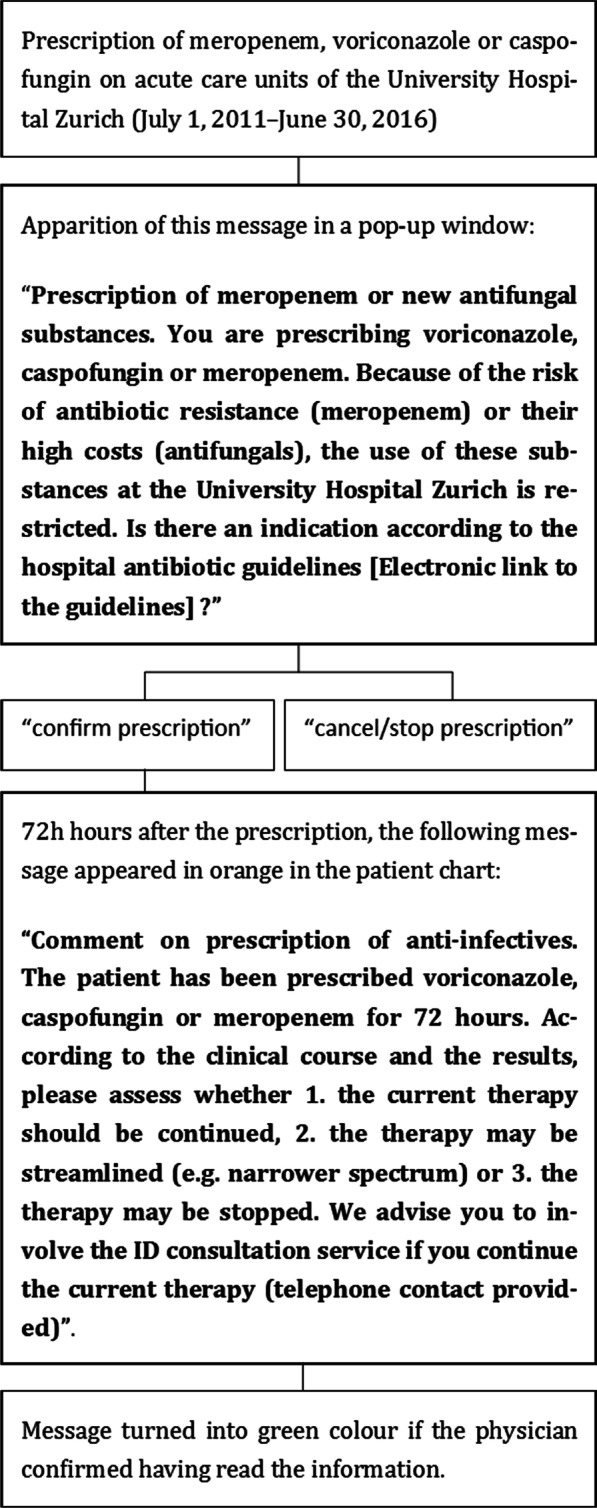
Intervention flow chart

The intervention consisted of the appearance of an alert in the patient’s electronic chart in case of prescriptions of meropenem, voriconazole or caspofungin (Fig. 1).

A pop-up window appeared for prescriptions in all acute care units from July 1, 2011, to June 30, 2016, and the following text was displayed: “Prescription of meropenem or new antifungal substances. You are prescribing voriconazole, caspofungin or meropenem. Because of the risk of antibiotic resistance (meropenem) or their high costs (antifungals), the use of these substances at the University Hospital Zurich is restricted. Is there an indication according to the hospital antibiotic guidelines? [Link to the guidelines provided]”. The physician then had to select “confirm prescription” or “cancel/stop prescription”. If prescription was confirmed, a horizontal bar appeared in the *warning/comment* section of the electronic patient chart 72 h after the prescription, displaying the following text: “Comment on prescription of anti-infectives. The patient has been prescribed voriconazole, caspofungin or meropenem for 72 h. According to the clinical course and the results, please assess whether (1) the current therapy should be continued, (2) the therapy may be streamlined (e.g. narrower spectrum) or (3) the therapy may be stopped. We advise you to involve the ID consultation service if you continue the current therapy (*telephone contact provided*)”. The bar was displayed in orange colour, indicating in our system that this information had to be validated by the treating physician, and turned into green colour if the physician confirmed having read the information.

### Data

Based on the Anatomical Therapeutic Chemical (ATC) Classification System, prescriptions of antibiotic groups ATC J01DH02 (meropenem, parenteral route) and antifungal groups J02AC03 (voriconazole, parenteral and oral) and J02AX04 (caspofungin, parenteral) for inpatients on acute wards (excluding intensive care units) were analyzed [16]. Antimicrobial prescriptions from July 1, 2010 to June 30, 2016 were included in the analyses.

The IT Department retrieved prescription data from electronic medical records. Prescription data contained (i) brand name, substance name, administration route and dose per unit of prescriptions of the substances meropenem, voriconazole and caspofungin, (ii) start and end of the prescription, (iii) birth date and age (in years) at admission of the patients, (iv) date of hospital admission and discharge and (v) date of admission to and discharge from the ward where the anti-infective substance was prescribed. We retrieved gender for each patient and number of bed days and number of admissions of all patients for each unit from the SAP® system [SAP (Suisse) SA, Bienne, Switzerland]. We defined 1 bed day as one bed occupied by one patient during 1 day and one night.

### Data analysis

We transformed the dataset using Python™ 2.7.6 (Python Software Foundation, Beaverton, OR, USA). We analyzed the data with the software R (R Core Team (2018). R foundation for Statistical Computing, Vienna, Austria) version 3.4.4 (Integrated Development for R. RStudio, Inc., Boston, MA, USA). We used the R packages *fpp* and *forescast* for all the time series analyses. R was also used for data edition and presentation.

### Study measurements

We measured drug prescription periods of meropenem, voriconazole and caspofungin as DOTs [8]. We aggregated DOTs per month and weighted DOTs for the number of bed days (DOTs/1000 bed days). In addition, we counted the number of prescriptions, defined as one uninterrupted prescription period of a drug, independently of the duration of each prescription. We aggregated the number of prescriptions per month and weighted them for the number of bed days (prescriptions/1000 bed days). In addition to the days and number of prescriptions of the three drugs, age, gender and length of stay were matched with prescription data.

### Longitudinal analysis of prescription data

We modeled the time trend and the impact of the alert on two outcome measures. Firstly, using interrupted time series regression, we assessed whether and in which magnitude the DOTs/1000 bed days changed after introduction of the alert. Secondly, we assessed whether and in which magnitude the number of prescription/1000 bed days changed after introduction of the alert. For both outcomes, we analyzed prescriptions of all three drugs together as well as the prescriptions of each drug independently.

### Seasonality

**Fig. 2:**
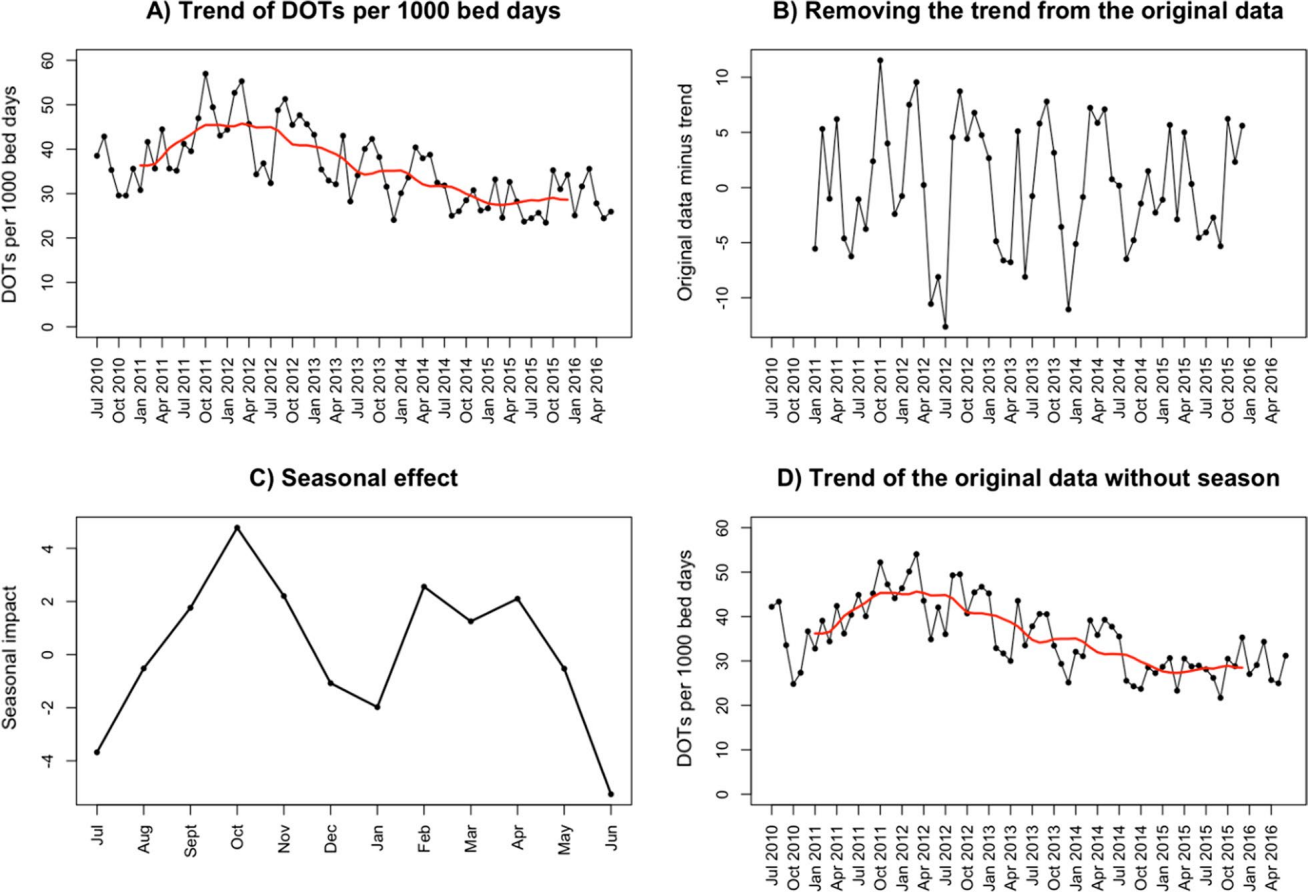
**A** The trend (red) of the original data: in this example days of therapy (DOTs) per 1000 bed days. **B** The original data (DOTs per 1000 bed days) minus the trend. **C** The seasonal effect was calculated based on the original data with the trend removed. **D** The seasonal effect was subtracted from the original data to obtain the trend (red) which is not influenced by seasonality

In order to account for the seasonal effect on drug prescriptions, we removed the seasonal component of the time series as follows: first, we performed a decomposition of the time series, i.e. the monthly prescription periods, into the trend, the seasonal (periodic, additive) effect and the remaining random effect. For this purpose, we determined the trend of the original data using the *ma* (simple moving average smoother) function of the R-package forecast (Fig. 2A). Second, we subtracted the trend from the original data (Fig. 2B). Third, we then calculated the mean of each month of the remaining data to estimate the seasonal effect (Fig. 2C). Thereafter, we removed the seasonal effect from the original data to assess the trend with exclusion of seasonality (Fig. 2D). The R-package *ts* (time series) was used for all calculations.

### Interrupted time series regression

To analyze the potential impact of the electronic alert on prescription periods, we analyzed the time trend of prescriptions of meropenem, voriconazole and caspofungin by using interrupted time series regression analysis. The analysis of the potential impact of the electronic alert was performed on the data with the seasonal effect removed. For this, we modelled the prescriptions before and after the introduction of the alert on July 1, 2011, by allowing a change in intercept and slope at this day. In particular, we used the segmented regression model

$$prescriptions\sim b_{0} *month + b_{1} *intervention + b_{2} *month*intervention,$$

where *month* indicates the month (numerical variable from 1 to 72) and *intervention* is a binary variable indicating whether the alert was present when the drug was prescribed or not. The coefficient *b*_*1*_ is the intercept and represents the immediate change in prescriptions after the introduction of the alert. The coefficient *b*_*2*_ is the change in slope of the regression after the introduction of the alert and represents the evolution of prescriptions over time.

## Results

### Study population

From July 1, 2010, until June 30, 2016, we obtained data covering a total of 1,771,543 bed days for all hospitalized patients, with a median of 24,364 bed days per month (IQR 22,901–25,653). The 4100 cases with prescription of one or more of the three substances accounted for 121,259 bed days in our institution (Table 1).

**Table 1:** Prescription data and study population

	Total	Meropenem	Voriconazole	Caspofungin
Duration and number of prescriptions				
Number of DOTs	64,281	39,156	12,640	12,485
DOTs per prescription period, median [IQR]	9 [5, 15]	8 [5, 14]	8 [4, 18]	11 [6, 18]
Number of prescription periods	5549	3695	1055	799
Study population				
Episodes	4100	3271	977	701
Length of stay, days	121,259	103,078	27,141	31,246
Length of stay, days, median [IQR]	23 [12, 38]	24 [14, 40]	24 [9, 36]	35 [19, 57]
Female sex, n (%)	1731 (42.2)	1367 (41.8)	441 (45.1)	311 (44.4)
Age on admission, years, median [IQR]	57 [43, 66]	57 [44, 67]	55 [43, 65]	53 [39, 63]

*DOTs* days of therapy, *IQR* interquartile range

The median length of stay (LOS) was 23 days [interquartile range (IQR) 12–38]. The LOS was longest with 35 days for patients who were prescribed caspofungin (IQR 19–57), followed by meropenem with 24 days (IQR 14–40) and voriconazole with 24 days (IQR 9–36). The median age at admission was 57 years (IQR 43–66). 42.2% of patients who were prescribed one or more substances were female, with similar proportions across each of the three substances.

### Descriptive analysis of DOTs and number of prescriptions

From July 1, 2010, until June 30, 2016, we counted 5549 prescriptions and 64,281 DOTS during 4100 hospital stays (Table 1). Meropenem accounted for 60.9% of all DOTs (39,156 DOTs, 3695 prescriptions), followed by voriconazole with 19.7% of all DOTs (12,640 DOTs, 1055 prescriptions) and caspofungin with 19.4% of all DOTs (12,485 DOTs, 799 prescriptions). The median duration of a prescription was 9 (IQR 5–15) days (caspofungin, 11 (IQR 6–18) days; meropenem, 8 (IQR 5–14) days; and voriconazole, 8 (IQR 4–18) days). In 3344 of 4100 cases (81.6%), only one substance was prescribed (meropenem alone in 2558 cases, voriconazole in 570 cases, and caspofungin in 216 cases). In 663 cases (16.2%) two substances were prescribed (meropenem and voriconazole in 271 cases, meropenem and caspofungin in 349 cases, voriconazole and caspofungin in 43 cases) and in 93 cases (2.3%) all three substances were prescribed simultaneously.

**Fig. 3:**
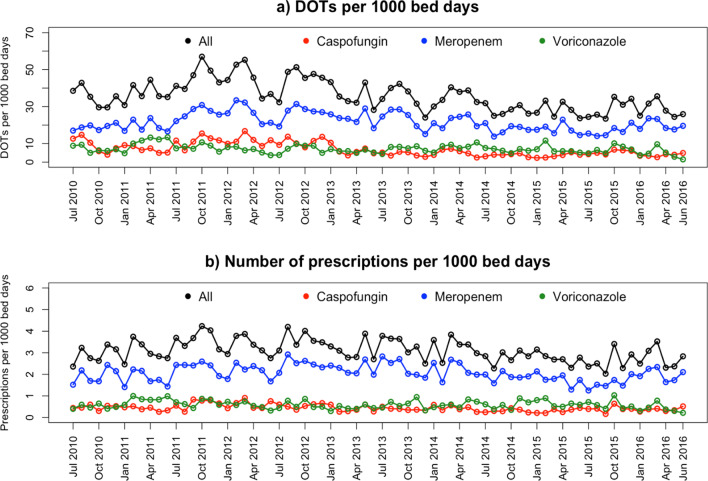
**A** The total number of days of therapy (DOTs) per 1000 bed days, total and for the three drug classes separately; **B** number of prescription periods per 1000 bed days, total and for the three drug classes separately

Overall, the median number of DOTs per 1000 bed days (including all hospitalized patients) per month was 34.7 (IQR 29.6–41.8) with a minimum of 23.5 in September 2015 and a maximum of 57.0 in October 2011 (Fig. 3A). The median DOTs/1000 bed days per month was 21.1 (IQR 17.9–25.5]) for meropenem prescriptions, 7.0 (IQR 5.2–8.6) for voriconazole and 5.6 (IQR 4.0–9.4) for caspofungin. The median number of prescriptions/1000 bed days was 3.1 (IQR 2.7–3.4) with a minimum of 2.0 in September 2015 and a maximum of 4.2 in October 2011 (Fig. 3B).

We observed a median of 2.1 (IQR 1.7–2.4) prescriptions/1000 bed days for meropenem, 0.6 (IQR 0.5–0.7) for voriconazole and 0.4 (IQR 0.3–0.5) for caspofungin.

### Interrupted time series regression

#### DOTs/1000 bed days

**Fig. 4:**
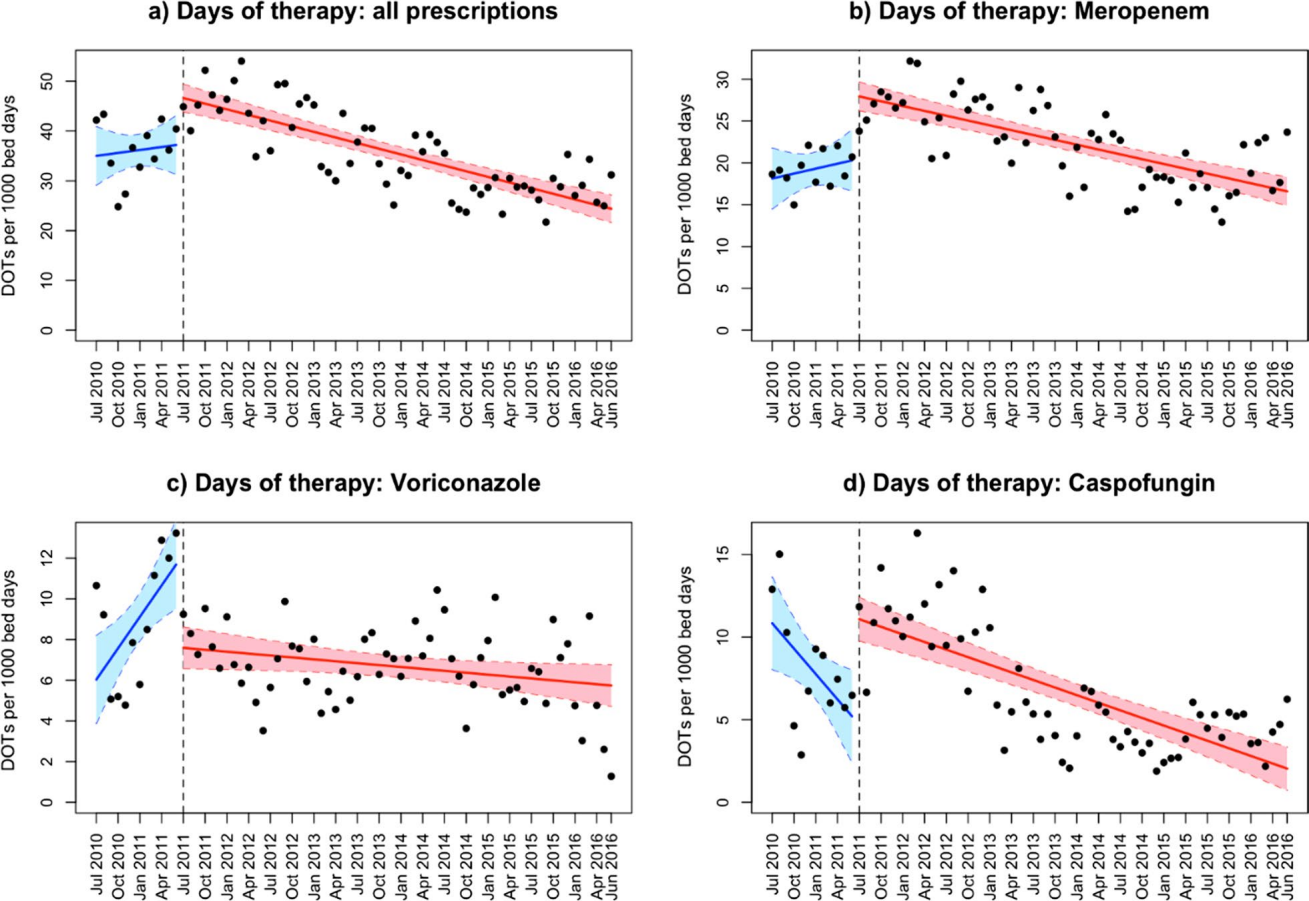
Interrupted time series regression analysis of days of therapy (DOTs) per 1000 bed days, for all substances combined and for the three single substances separately

There was no significant change (− 0.58, 95% CI − 1.48–0.33, *P* = 0.2) in the trend of prescriptions (slope of DOTs) when comparing the time before the alert, i.e., July 2010 until June 2011, with the time after the alert, i.e., July 2011 until June 2016 (Table 2 and Fig. 4).

**Table 2:** Results of the regression analysis of DOTs per 1000 bed days and number of prescriptions per 1000 bed days

Variable	All drugs	Meropenem	Voriconazole	Caspofungin
DOTs/1000 bed days [95% CI]				
Intercept pre-interv.	37.38 [30.76, 44.0]	20.49 [16.36, 24.61]	12.2 [9.75, 14.64]	4.7 [1.53, 7.86]
Change of intercept	9.23 [2.07, 16.40]	7.45 [3.00, 11.90]	− 4.61 [− 7.26, − 1.96]	6.39 [2.97, 9.82]
*P* intercept	0.0123	0.0014	< 0.001	< 0.001
*Slope pre-intervention*	0.2 [− 0.7, 1.1]	0.2 [− 0.36, 0.76]	0.51 [0.18, 0.85]	− 0.51 [− 0.94, − 0.08]
Change of slope	− 0.58 [− 1.48, 0.33]	− 0.39 [− 0.95, 0.17]	− 0.55 [− 0.88, − 0.21]	0.36 [− 0.07, 0.79]
*P* slope	0.2079	0.1713	0.0017	0.1018
Prescriptions per 1000 bed days [95% CI]				
Intercept pre-interv.	3.28 [2.78, 3.78]	1.93 [1.52, 2.34]	0.97 [0.77, 1.17]	0.38 [0.21, 0.54]
Change of intercept	0.37 [− 0.17, 0.92]	0.52 [0.08, 0.97]	− 0.36 [− 0.58, − 0.14]	0.21 [0.03, 0.39]
*P* intercept	0.1738	0.0211	0.0014	0.0204
*Slope pre-intervention*	0.05 [− 0.02, 0.12]	0.01 [− 0.05, 0.06]	0.05 [0.02, 0.07]	− 0.01 [− 0.03, 0.01]
Change of slope	− 0.07 [− 0.13, 0.00]	− 0.02 [− 0.08, 0.04]	− 0.05 [− 0.08, − 0.02]	0 [− 0.02, 0.03]
*P* slope	0.0619	0.4724	< 0.001	0.7092

*CI* confidence interval, *DOTs* days of therapy, *interv.* intervention

When analyzing prescriptions of the three drugs of interest separately, only DOTs/1000 bed days for voriconazole revealed a significant decrease in slope (*P* = 0.0017) after the introduction of the alert. Noteworthy, the regression intercept was significantly higher than 0 (*P* = 0.01) when analyzing DOTs/1000 bed days for all drugs combined, as well as for meropenem (*P* = 0.0014) and caspofungin (*P* < 0.001), meaning that prescriptions of these drugs increased significantly immediately after introduction of the alert. Only in the case of voriconazole, the intercept was significantly lower than 0 (*P* < 0.001) after the introduction of the alert.

#### Prescriptions/1000 bed days

**Fig. 5:**
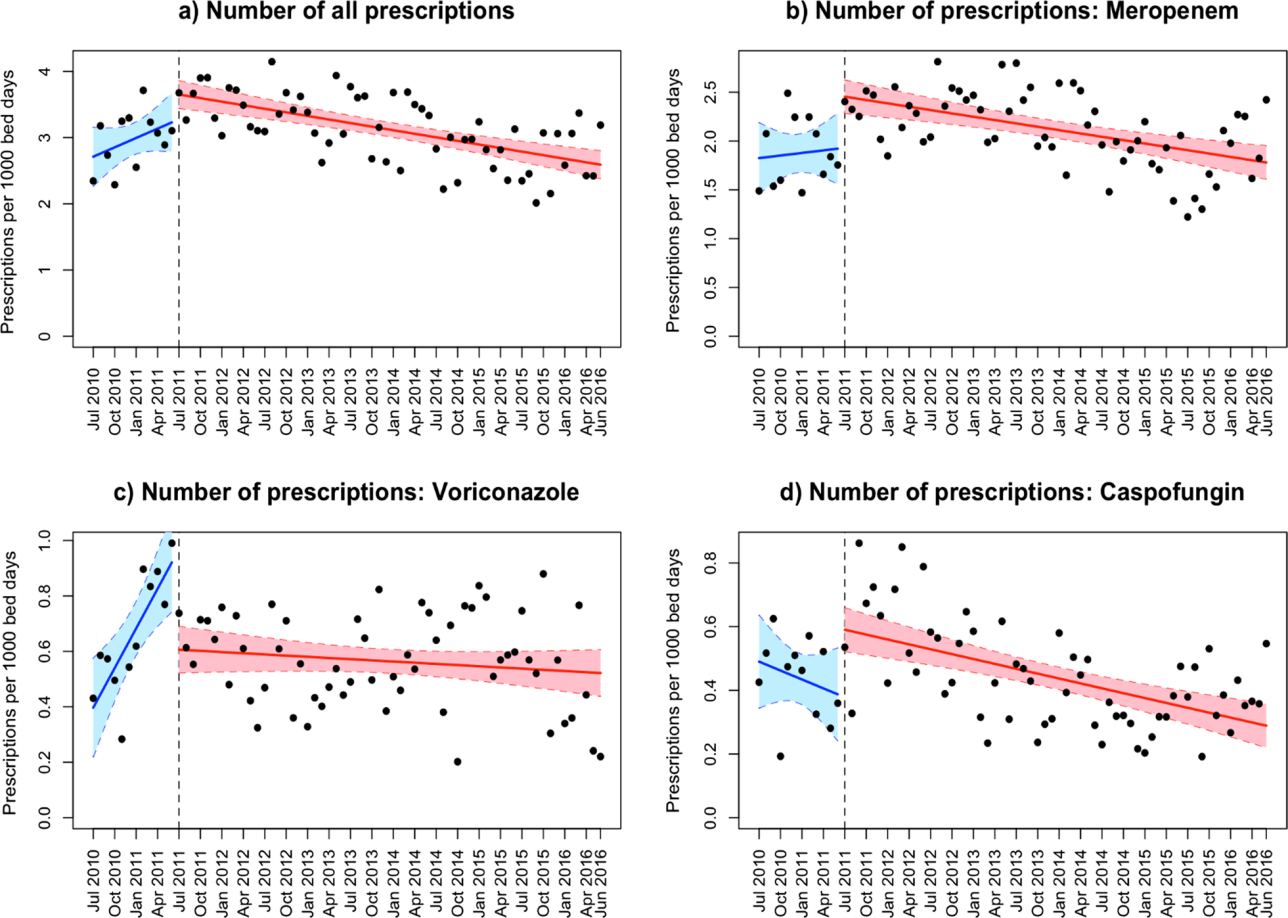
Interrupted time series regression analysis of the number of prescriptions per 1000 bed days, for all substances combined and for the three single substances separately

Similar to the previous analysis, there was no significant change (*P* = 0.062) in slope when analyzing all drug classes combined. Again, only the slope of voriconazole prescriptions per 1000 bed days decreased significantly (*P* < 0.001) after the introduction of the alert (Table 2 and Fig. 5). Changes in the intercept were in line with the analysis of DOTs/1000 bed days.

## Discussion

This study examined the effect of an electronic alert on duration and number of prescriptions of anti-infective substances. Overall, the duration and the number of prescriptions tended to decrease after introduction of the alert, but the effect was not statistically significant. The stratified analysis of the individual drugs showed that only the prescriptions of voriconazole decreased significantly after the intervention.

The reduction of the duration as well the number of prescriptions of voriconazole is an encouraging signal supporting review of prescriptions by prescribers themselves [8]. Whether the alert alone, further non-measured interventions or a decreasing incidence in invasive fungal infections, led to this reduction, cannot be assessed due to the study design and the limited data available. Furthermore, the ASP at our institution is involved in many interventions, which possibly also explain the reduction of voriconazole prescriptions (ID consultations, ID visits on intensive care units, local guidelines, educational programs). Epidemiological reasons, such as increasing rates of multidrug-resistant gram-negative bacteria or an increasing incidence of invasive or azole-resistant *Candida* infections, on the other hand, may have concealed a true intervention effect for meropenem or caspofungin [11, 17]. As voriconazole is mainly used for the treatment of invasive *Aspergillus fumigatus* infections, such an opposite trend may not have played a major role for this substance.

Although the message that was provided in the alert clearly suggested to strongly re-consider the indication of the therapy, it may not have persuaded the treating physicians to stop the therapy or to contact the ID team. The direct effect of the alert on the number of ID consultations was not analyzed in this study but is an outcome of interest for further studies.

Our results are consistent with a previous publication from Lesprit et al. [18] In a similar study, the investigators could show that distribution of an information sheet advising an adaptation of treatment and a questionnaire at day 4 of an i.v. antibiotic therapy did not influence prescriptions of 13 selected i.v. antibiotics, in comparison to the control group. In the group with an additional ID consultation, prescriptions were significantly modified after day 4. Similarly, a previous study in the intensive care setting of a tertiary hospital in Chicago has shown a clear benefit of checklist and face-to-face prompting of physicians on antibiotic duration and risk-adjusted mortality in ICU patients receiving antibiotics, when compared to a checklist alone [19]. Prompting may be seen as part of prospective audit and feedback. Such interventions catalyze contacts between physicians and the ID advising team, which may be much more effective than self-stewardship [20, 21]. Prospective audit and feedback require available ID physicians and the resources for such a support are oftentimes lacking. ASP in our institution is based on ID consultation (on demand), ID visits in ICUs, and publications of guidelines, reports on consumption of anti-infective substances and bacterial resistance patterns.

An additional preauthorization by an ID physician, which is a more drastic intervention, would have possibly led to a stronger effect of the message provided [8]. However, the medical direction in Swiss hospitals rarely enforces preauthorization for antibiotics. This is possibly due to a strong prescriber autonomy in Switzerland. The absence of a patient, disease or substance specific education, further key interventions of ASPs, may also have led to the lack of statistical difference in this intervention study [8]. Furthermore, the psychological effect of the alert on the prescribing physicians may have vanished over time, as seen in other studies with electronic stewardship interventions, although such a pattern could not be confirmed in our data [22].

Finally, targeting only the prescribing physicians may have reduced the potential impact of the alert. Prescribing physicians are mostly fellows in training, who due to lack of time, motivation or knowledge, may underestimate the importance of the optimization of anti-infective prescriptions. One the other hand, the choice of the anti-infective substance is almost always made by the residents or specialists and rather not fellows.

Strengths of this study are the large number of patients, the long observation period and the use of prescription data. The study has also several limitations. We cannot explain why we observed a sudden increase of meropenem and caspofungin prescriptions shortly after the alert was introduced. The message provided at and 72 h after prescription advised to re-evaluate the choice of the substance or to contact the ID team. The quality of the dataset depends on the electronic prescription system, which is in a constant modification state. It is possible that the alert introduction led to an optimization of data and thus captured more prescriptions. One other hypothesis is that the ID counselling service was more often involved, and indeed decided to lengthen or re-introduce prescriptions, since the chosen antibiotics seemed justifiable to the ID team. Third, we do not collect additional clinical data of included patients, which clearly exclude further explorative analyses. Also, structural changes have certainly occurred during the study period, which have not been assessed in this analysis but may well have influenced the results observed. Furthermore, data on other broad-spectrum antibiotics were not analyzed. This prevents us to assess whether the intervention led to switches to other broad-spectrum antibiotics, not covered by the alert and thus escaping our surveillance. Last, generalizability to other settings is limited since data originates from a single, tertiary care centre.

## Conclusions

The introduction of an electronic reminder for prescriptions of meropenem, voriconazole and caspofungin led to a significant decrease of voriconazole prescriptions in comparison with the pre-intervention period, but did not consistently alter prescription patterns of meropenem or caspofungin. According to the results and other available evidence, self-stewardship supported by electronic reminders may only have a limited impact on prescriptions of anti-infective substances. Self-stewardship may thus benefit from face-to-face prompting with ID physicians and audit and feedback.

## Data Availability

The datasets generated during and analysed during the current study are not publicly available due to confidentiality but are available from the corresponding author on reasonable request.

## Abbreviations

ASP: Antimicrobial stewardship program
ATC: Anatomical Therapeutic Chemical
DDD: Defined daily doses
DOTs: Days of therapy
ID: Infectious disease
IDSA: Infectious Diseases Society of America
IQR: Interquartile range
LOS: Length of stay
SHEA: The Society for Healthcare Epidemiology of America
